# Carbazole-functionalized hyper-cross-linked polymers for CO_2_ uptake based on Friedel–Crafts polymerization on 9-phenylcarbazole

**DOI:** 10.3762/bjoc.15.279

**Published:** 2019-11-26

**Authors:** Dandan Fang, Xiaodong Li, Meishuai Zou, Xiaoyan Guo, Aijuan Zhang

**Affiliations:** 1 School of Materials Science & Engineering, Beijing Institute of Technology, Beijing 100081, China

**Keywords:** 9-phenylcarbazole, CO_2_ uptake, Friedel–Crafts polymerization, hyper-cross-linked polymers, microporous

## Abstract

To systematically explore the effects of the synthesis conditions on the porosity of hyper-cross-linked polymers (HCPs), a series of 9-phenylcarbazole (9-PCz) HCPs (**P1**–**P11**) has been made by changing the molar ratio of cross-linker to monomer, the reaction temperature *T*_1_, the used amount of catalyst and the concentration of reactants. Fourier transform infrared spectroscopy was utilized to characterize the structure of the obtained polymers. The TG analysis of the HCPs showed good thermal stability. More importantly, a comparative study on the porosity revealed that: the molar ratio of cross-linker to monomer was the main influence factor of the BET specific surface area. Increasing the reaction temperature *T*_1_ or changing the used amount of catalyst could improve the total pore volume greatly but sacrificed a part of the BET specific surface area. Fortunately changing the concentration of reactants could remedy this situation. Slightly changing the concentration of reactants could simultaneously obtain a high surface area and a high total pore volume. The BET specific surface areas of **P3** was up to 769 m^2^ g^−1^ with narrow pore size distribution and the CO_2_ adsorption capacity of **P11** was up to 52.4 cm^3^ g^−1^ (273 K/1.00 bar).

## Introduction

HCPs get more attention in recent years due to their high BET specific surface area [1], made under mild reaction conditions [2], used nonprecious materials as catalyst [3] and wide applications [4–9], etc. The synthesis methods of HCPs include solvent knitting methods [10], Scholl coupling reaction [11], the knitting method with formaldehyde dimethyl acetal (FDA) [12], functional group reactions [2,13] and so on. Among these methods, the knitting method with FDA as external cross-linker is the most time-efficient approach [14]. FDA was first used as cross-linker to knit aromatic building blocks [15]. Researchers used this method to knit triptycenes [16], triphenylphosphine [4], benzimidazole, 1,3,5-triphenylbenzene [17], carbazole [18], naphthol-based monomers [10] etc. with FDA to obtain various HCPs, which exhibited outstanding porous properties.

Studies of the effects on porosity of HCPs are significant, but almost all investigations focused on the role of monomer length and geometry on the porosity [6,19–32]. Researchers have synthesized a series of carbazole-based microporous HCPs and came to the conclusion that 2D and 3D-conjugated architectures with nonplanar rigid conformation and dendritic building blocks were favorable for getting a high BET specific surface area [6,19–21,23–25]. Qiao synthesized five microporous materials using carbazole with different flexible chains, proving that the flexible chain length was an important factor for the porosity [26]. Different phenyl-based structures were also synthesized to explore the effect of the monomer structure on the porosity [27–32]. However, researchers seldom cared about the effect of reaction conditions on the porosity, which is of far-reaching significance in preparation of HCPs.

In this work, a series of HCPs was synthesized from 9-PCz with FDA as the external cross-linker, the porosity was tuned by variation of the reaction conditions such as the molar ratio of cross-linker to monomer, the reaction temperature *T*_1_, the amount of used catalyst and the concentration of reactants. Additionally, the CO_2_ uptake of the obtained polymers was explored.

## Results and Discussion

**Scheme 1:**
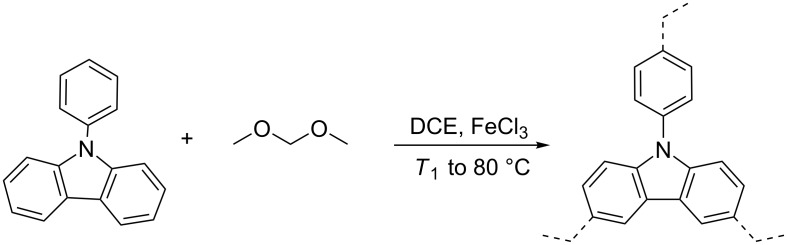
Synthetic route to HCPs.

The synthesis of HCPs is shown in Scheme 1 and Table 1. Using the Friedel–Crafts reaction, 11 samples (**P1**–**P11**) have been synthesized. To study the effect of the synthesis conditions on the porosity the molar ratio of building unit to cross-linker (**P1**–**P5**), the reaction temperature *T*_1_ (**P3**, **P6**, **P7**), the amount of the catalyst used (**P3**, **P8**, **P9**) and the concentration of reactants (**P3**, **P10**, **P11**) were varied.

**Table 1:** Reaction conditions for the preparation of HCPs.

HCPs	9-PCz [mmol]	FDA/9-PCz	FeCl_3_ [g]	FDA concentration [mol L^−1^]	*T*_1_ [°C]

**P1**	2	1	0.64	0.11	rt
**P2**	1	2	0.64	0.11	rt
**P3**	0.67	3	0.64	0.11	rt
**P4**	0.5	4	0.64	0.11	rt
**P5**	0.4	5	0.64	0.11	rt
**P6**	0.67	3	0.64	0.11	40
**P7**	0.67	3	0.64	0.11	50
**P8**	0.67	3	0.48	0.11	rt
**P9**	0.67	3	0.80	0.11	rt
**P10**	0.67	3	0.64	0.13	rt
**P11**	0.67	3	0.64	0.10	rt

### Chemical structure analysis

**Figure 1:**
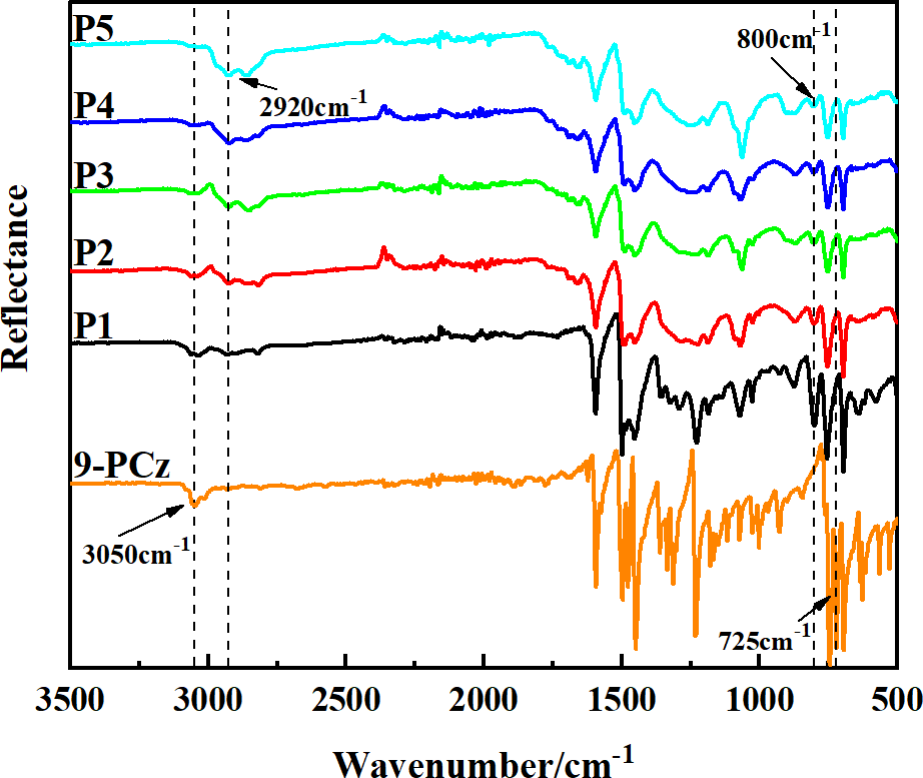
FTIR spectrum of HCPs **P1**–**P5** and 9-PCz.

**Figure 2:**
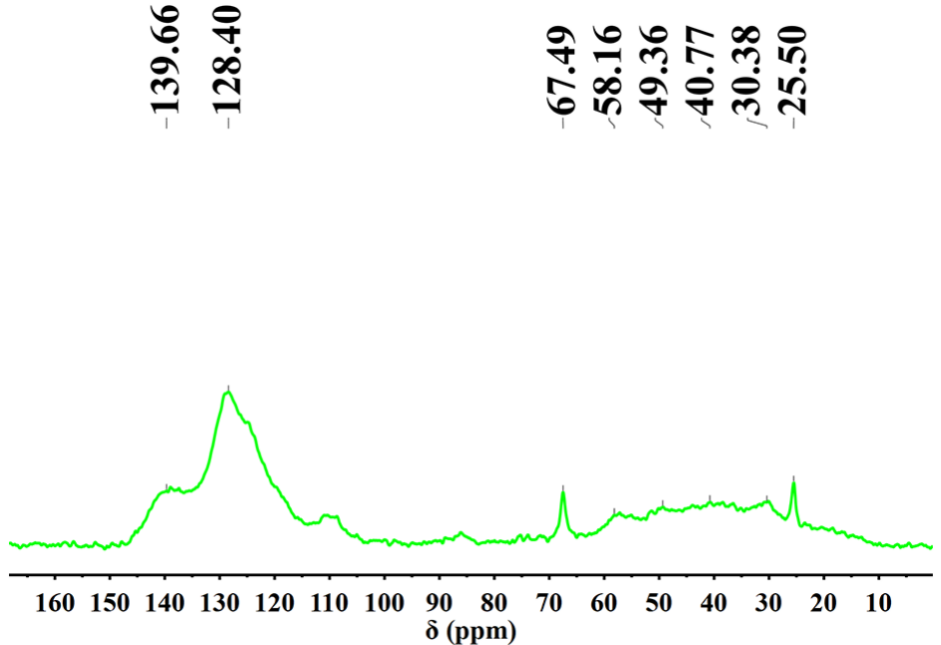
Solid state ^13^C NMR spectrum of **P3**.

FTIR spectra were measured to verify the structure of HCPs (Figure 1). The peak at 3100–3000 cm^−1^ correspond to the C–H stretching vibrations of the aromatic rings, which declined obviously in **P1**–**P5** compared to monomer 9-PCz. The peak of the disubstituted phenyl ring in the 9-PCz monomer at near 725 cm^−1^ disappeared while the peak of the trisubstituted phenyl ring near 800 cm^−1^ was dominant in polymers [20]. C–H stretching vibration at about 2920 cm^−1^ belongs to the structure of -CH_2_- in the HCPs [9,33]. The FTIR spectra of **P6**–**P11** (Supporting Information File 1, Figure S1) were similar to the ones for **P1**–**P5**. **P1**–**P11** are polymers with very similar chemical structure, which have been proved by FTIR. In addition, we performed solid state ^1^H NMR and solid state ^13^C NMR on **P3** as a representative sample. The solid state ^1^H NMR showed peaks in the range of 1.5–3.5 ppm for the saturated protons (Supporting Information File 1, Figure S2). Also, the solid state ^13^C NMR (Figure 2) showed peaks between 25–50 ppm, indicating sp^3^ carbons [9,16]. The peaks about 139 ppm belong to the substituted aromatic carbon, the peaks about 128 ppm were attributed to the unsubstituted aromatic carbon. Based on the above peaks in the solid state NMR, the Friedel–Crafts polymerization product was confirmed.

### TGA analysis

**Figure 3:**
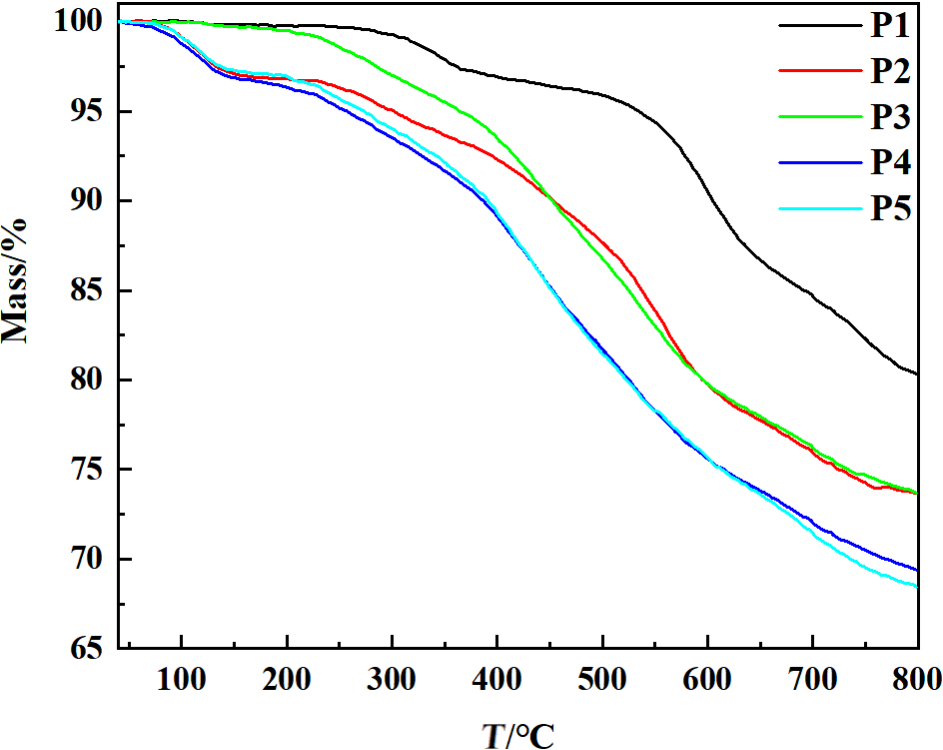
TGA curves of HCPs **P1**–**P5**.

The thermal stability of HCPs was investigated by TGA tests (Figure 3 and Supporting Information File 1, Figure S3). A slight weight loss at 100 °C was observed for **P2**, **P4**, **P5**, and **P7**, due to the solvent wrapped in the hyper-cross-linked networks, which could not be removed even in vacuum [16]. Except this, the TGA curves of **P1**–**P11** exhibited similar decomposition behavior. The highest decomposition temperature of **P1**–**P11** was up to 594 °C, with ca. 70% mass residues even when the temperature raised up to 800 °C (Supporting Information File 1, Table S1), demonstrated the splendid thermal stability of **P1**–**P11** as reported for microporous polymers [34].

### Morphology analysis

**Figure 4:**
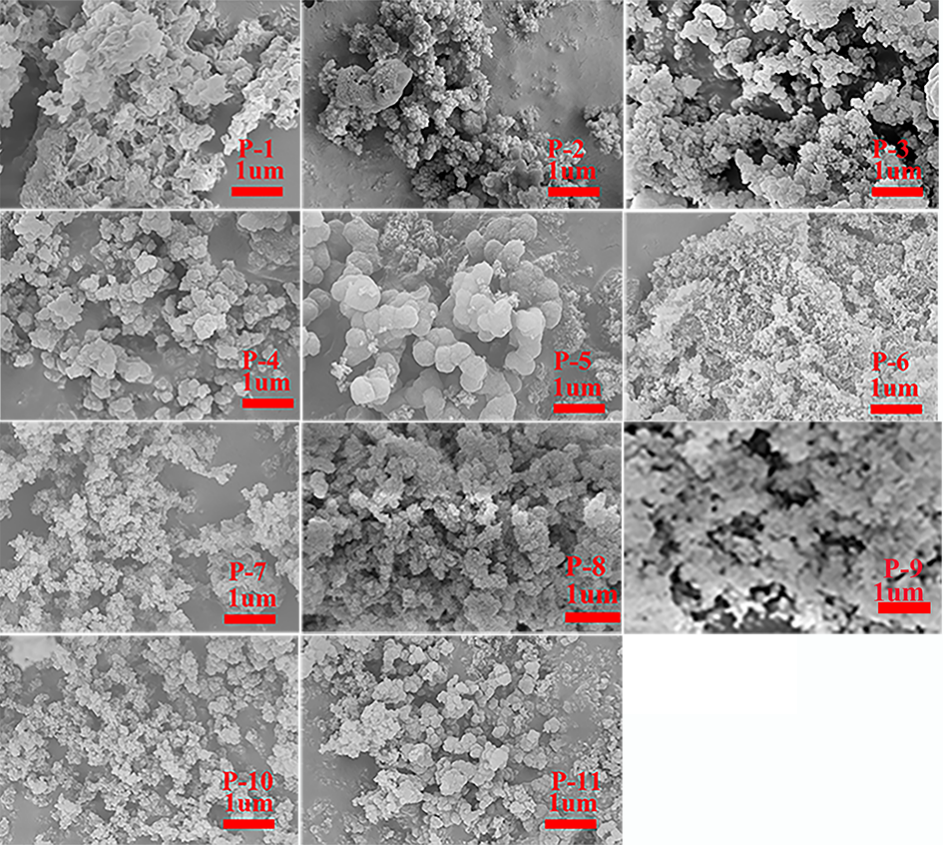
Scanning Electron micrograph of HCPs.

**Figure 5:**
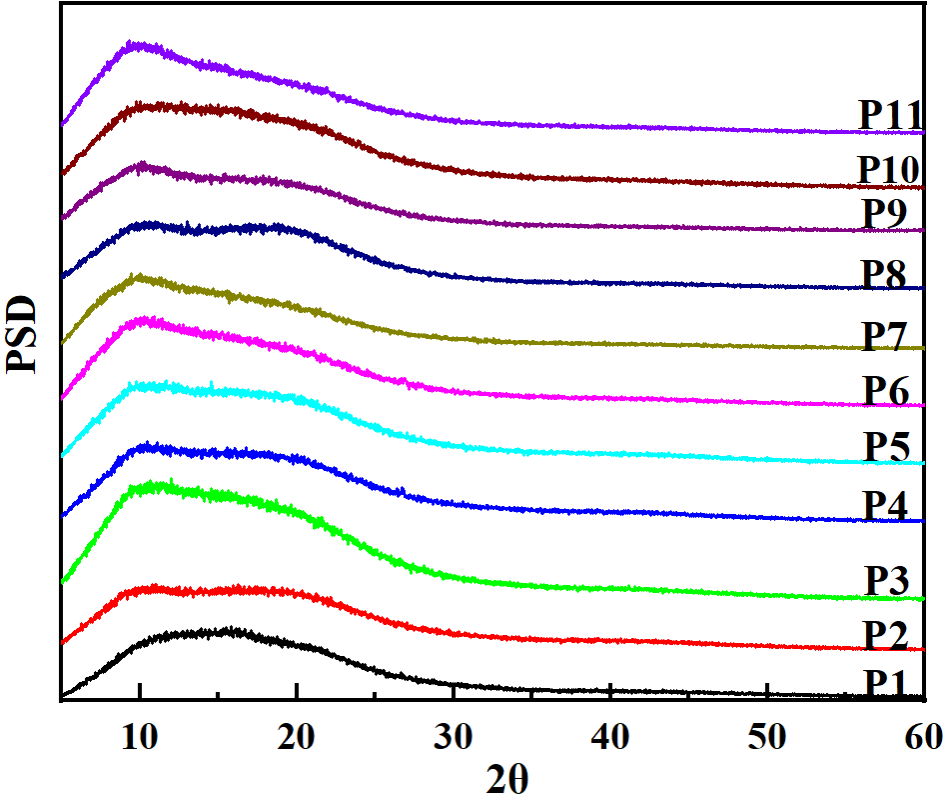
The XRD curves of HCPs.

The morphology of **P1**–**P11** was investigated by SEM images (Figure 4), which showed that HCPs were composed of rough surface particles. The particles had different size and agglomerated to loose aggregates. There were plentiful pores randomly distributed among the particles. X-ray diffraction (XRD) of obtained HCPs exhibited similar diffraction patterns, only a round peak at 10°, hinting that **P1**–**P11** were amorphous polymers [12] (Figure 5).

### Porous properties

The permanent porous nature was subsequently studied by subjecting the polymers to nitrogen adsorption–desorption experiments at 77 K. The porosity data of **P1**–**P11** are listed in Table 2. The main influence of the porous properties is the reaction degree, as well as the C/N ratio, which has been confirmed by elemental analysis. Elemental analyses were measured to compare the degree of crosslinking of **P1**–**P11** (Table 3), because the C/N ratio of the polymer will increase as the degree of crosslinking increases.

**Table 2:** Porosity data for HCPs.

HCPs	S_BET_^a^ [m^2^ g^−1^]	S_micro_^b^ [m^2^ g^−1^]	SA_lang_^c^ [m^2^ g^−1^]	V_total_^a^ [cm^3^ g^−1^]	V_micro_^d^ [cm^3^ g^−1^]

**P1**	350	0	481	0.47	0
**P2**	709	311	970	0.62	0.14
**P3**	769	331	1049	0.63	0.14
**P4**	696	322	926	0.59	0.14
**P5**	721	319	965	0.76	0.14
**P6**	659	286	880	1.24	0.13
**P7**	599	271	800	1.12	0.12
**P8**	612	328	821	0.64	0.15
**P9**	671	336	890	1.00	0.15
**P10**	755	334	1013	1.11	0.15
**P11**	760	343	1025	1.27	0.15

^a^Surface area and pore volume were obtained by the Brunauer–Emmett–Teller (BET) method in the pressure range of 0.05–0.35 *P*/*P*_0_, the standard deviation of the porosity is 0.1%; ^b^microporous surface area calculated from the adsorption branch of the nitrogen adsorption-desorption isotherm using the *t*-plot method; ^c^surface area calculated from the nitrogen adsorption branch based on the Langmuir model; ^d^microporous volume calculated from the adsorption branches using NLDEF methods.

**Table 3:** Elemental analysis data of HCPs.

HCPs	N%	C%	H%	C/N

**P1**	5.64	83.83	4.74	14.87
**P2**	4.43	80.31	4.87	18.15
**P3**	3.93	77.75	4.90	19.79
**P4**	3.63	77.29	5.04	21.27
**P5**	3.47	77.46	5.09	22.32
**P6**	4.05	78.20	4.97	19.31
**P7**	4.17	80.13	5.07	19.20
**P8**	4.37	81.20	5.14	18.58
**P9**	4.13	77.51	4.98	18.78
**P10**	4.32	78.50	4.69	18.18
**P11**	4.30	78.22	4.65	18.18

### The effect of molar ratio of cross-linker to monomer on the porosity of HCPs (**P1–P5**)

**Figure 6:**
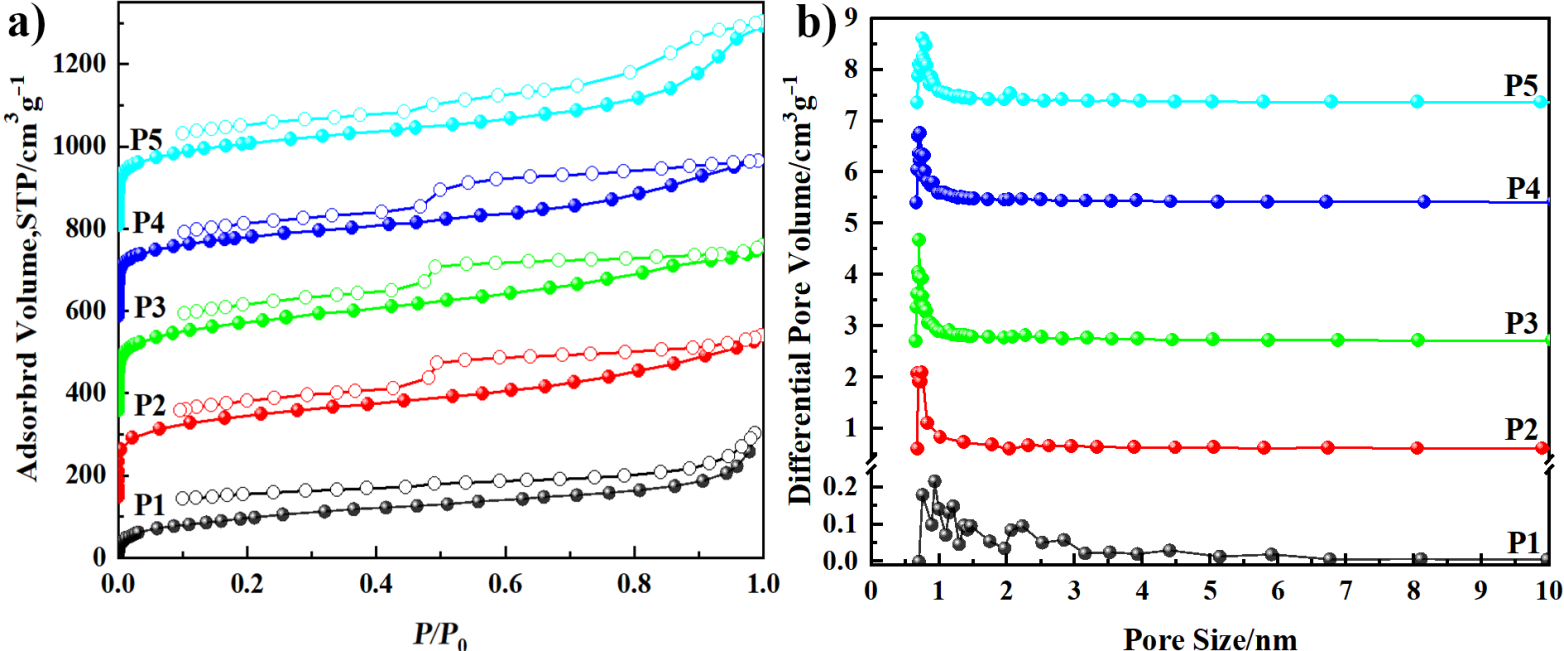
Nitrogen sorption isotherms (a) and pore size distribution (b) of **P1**–**P5**.

To explore the effect of molar ratio of cross-linker to monomer on the porosity of HCPs, five polymers (**P1**–**P5**) were synthesized. The reaction conditions of **P1**–**P5** were similar except the gradually increasing molar ratio of FDA to 9-PCz from 1 to 5. Except **P1**, **P2**–**P5** exhibited a rapid nitrogen adsorption ability at low pressures (*P*/*P*_0_ < 0.05, Figure 6a), which indicated that the micropores exist in the networks [35]. The sorption isotherm of **P2**–**P4** exhibited a combination of type I and IV nitrogen sorption isotherms according to the IUPAC classification [36]. The hysteresis between adsorption and desorption of **P2**–**P4** indicates that the polymers contain mesopores [37]. There is no sharp rise at high relative pressures (*P*/*P*_0_ > 0.9) of **P1**–**P5**, which means that scarcely macropores exist in the networks [38]. The pore size distribution (Figure 6b) was calculated from the adsorption branches using nonlocal density functional theory (NLDFT) methods. **P2**–**P5** exhibited narrow pore size distribution in the micropore scopes (<2 nm), while **P1** showed a wide pore size distribution. The surface areas of **P1**–**P5** ranged from 350 to 769 m^2^ g^−1^ (Table 2). The lowest specific surface area of **P1** was due to the less FDA which reduced the crosslinking density. Largely exaltation of BET surface area from **P1** to **P3** was due to the increasing FDA/9-PCz ratio improved the crosslinking density which could be confirmed by the increasing C/N ratio (Table 3). However, further increasing the molar ratio of FDA to 9-PCz could not result in a higher BET specific surface area, because the high steric hindrance prevented further crosslinking reaction [39], the raised C/N ratio (Table 3) maybe because of the tail end groups of FDA. The BET specific surface area of **P3** was much higher than the polymer CZB (with similar carbazole monomer) [18]. All that reveal that enough and suitable cross-linker amount was the premise of superior specific surface areas.

### The effect of reaction temperature *T*_1_ on the porosity of HCPs (**P3**, **P6**, **P7**)

The reaction temperature can mainly influence the polymerization process; to explore the effect of the reaction temperature on the porosity, we have synthesized **P6** and **P7** by increasing the reaction temperature *T*_1_ from rt (**P3**) to 40 and 50 °C. When comparing the sorption isotherms of the HCPs (Supporting Information File 1, Figure S4), it was envisioned that those of **P6** and **P7** were a combination of type I and II nitrogen sorption isotherms, which emerged two steep N_2_ adsorption abilities at the low pressure region (*P*/*P*_0_ < 0.1) and the high pressure region (*P*/*P*_0_ > 0.9), indicating that micropores and macropores appeared in the polymers [40]. The BET specific surface area of **P3**, **P6** and **P7** (769 m^2^ g^−1^, 659 m^2^ g^−1^ and 599 m^2^ g^−1^, respectively) decreased with increasing the reaction temperature *T*_1_, microporous surface area and the microporous volume presented the same trend. According to the C/N ratio, the crosslinked degree of **P3** is also better than **P6**, **P7**. But the total pore volume (0.63 cm^3^ g^−1^, 1.24 cm^3^ g^−1^ and 1.12 cm^3^ g^−1^, respectively) increased heavily with increasing *T*_1_. The total pore volume of **P6** and **P7** is much bigger than the carbazole-based HCPs such as CPOP-13 (890 m^2^ g^−1^, 0.468 cm^3^ g^−1^), CPOP-14 (820 m^2^ g^−1^, 0.416 cm^3^ g^−1^), Cz-POF-4 (914 m^2^ g^−1^, 0.6 cm^3^ g^−1^) [20,33]. This may be the result of that excessive temperature caused excessive crosslink at the beginning of the reaction, the plethora network cocooned a part of the reaction center, and prevented it from further cross-linking (micropores), hence it formed macropores. All that indicated that improving the reaction temperature *T*_1_ could enhance the total pore volume of HCPs but lowers the specific surface area.

### The effect of the amount of catalyst on the porosity of HCPs (**P3**, **P8**, **P9**)

To explore the effect of the amount of catalyst on the HCPs porosity, **P8** (3 mmol FeCl_3_), **P3** (4 mmol FeCl_3_), **P9** (5 mmol FeCl_3_) were made by varying the catalyst amount. The nitrogen sorption isotherms (Supporting Information File 1, Figure S5) of **P8** and **P9** were similar as the one of **P7**; this means that micropores and macropores exist simultaneously in the polymers. The specific surface area for **P8** (612 m^2^ g^−1^) and **P9** (671 m^2^ g^−1^) was inferior to **P3** (769 m^2^ g^−1^), this trend was similar to the C/N ratio. The total pore volume of **P9** (1 cm^3^ g^−1^) was much higher than **P3** (0.63 cm^3^ g^−1^), **P8** (0.64 cm^3^ g^−1^). The influence of the amount of catalyst used on the porosity can result in a high specific surface area, when applying a suitable amount of catalyst.

### The effect of the concentration of reactants on the porosity of HCPs (**P3**, **P10**, **P11**)

The effect of the concentration of reactants on the porosity was studied by changing the FDA concentration in the synthesis of HCPs **P3**, **P10**, and **P11**. As shown in Supporting Information File 1, Figure S6 and in Table 2, the sorption isotherms of **P10** and **P11** are similar to the one of **P7**, which signifies the presence of permanent micropores and macropores in the polymers [41–44]. While the **P3** porosity was composed of micropores and mesopores as above-mentioned, the pore size distribution of the three HCPs were similar and showed a narrow distribution in the micropores region and a pore size center at ca. 0.7 nm. The BET specific surface areas of **P3**, **P10**, and **P11** were about the same (769 m^2^ g^−1^, 755 m^2^ g^−1^, 760 m^2^ g^−1^, respectively), the microporous surface area and the microporous volume were also similar. However, there were wide disparities in the total pore volume among the obtained polymers. The volume of **P10** (1.11 cm^3^ g^−1^), **P11** (1.27 cm^3^ g^−1^) was about twice that of **P3** (0.63 cm^3^ g^−1^) which is owing to the extra generated macropores, and it is higher than many carbazole-based HCPs with similar BET specific surface area [19–20,24]. We conjecture that the concentration of FDA can affect the formation process and morphology of the polymers, when polymer particles agglomerate and stack together (Figure 4). We reached the conclusion that when varying the concentration of reactants slightly, a great increase of the pore volume can be accomplished without sacrificing the BET special surface area.

#### CO_2_ uptake behavior

**Figure 7:**
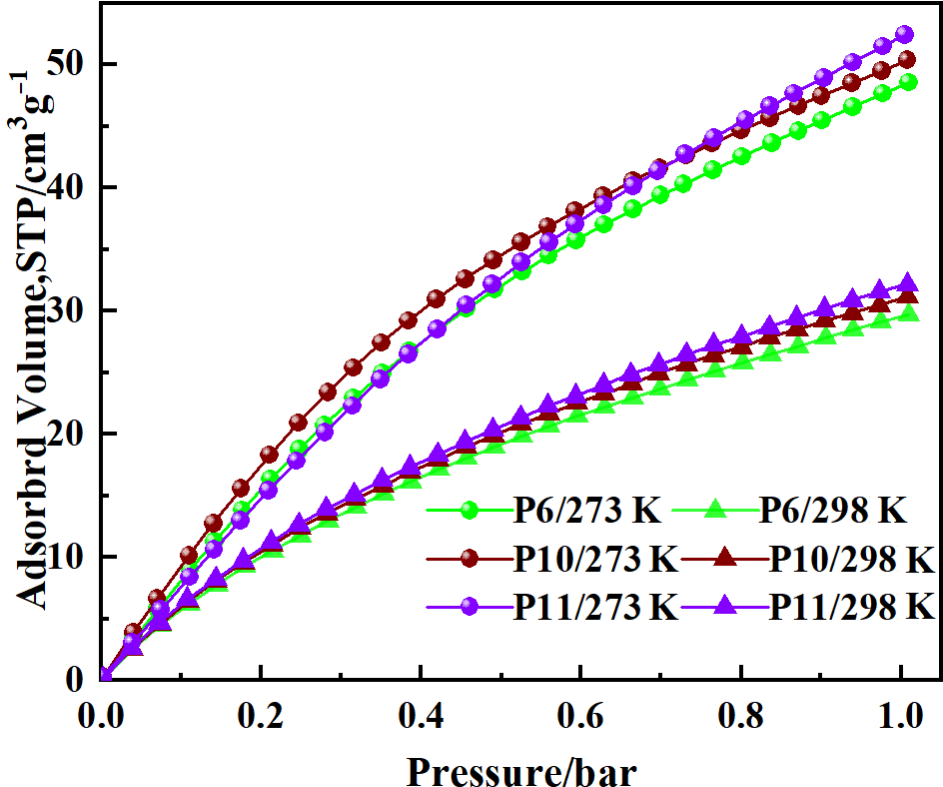
Volumetric CO_2_ adsorption isotherms up to 1 bar of **P3**, **P10**, and **P11**.

The presence of many CO_2_-philic sites (N-bearing substituents) and narrow pore distribution in the networks could improve the molecular interaction with CO_2_ [18]. Hence, three polymers (**P3**, **P10**, and **P11**) were selected as representative samples to conduct CO_2_ adsorption experiments up to 1 bar at both 273 and 298 K (Figure 7). HCPs showed a similar and moderate CO_2_ uptake (Table 4). **P11** displayed the optimal CO_2_ storage of 52.4 cm^3^ g^−1^ (10.4 wt %) at 1.0 bar/273 K. which was higher than that of the carbazole-based microporous polymers PBT-C1 (46 cm^3^ g^−1^) [26], CMPSO-1B3 (46.8 cm^3^ g^−1^) [24], CPOP2-4 (7.8–9.0 wt %) [19], tetraphenylmethane-based CPOP10 (S_BET_ = 3337 m^2^ g^−1^, 9.1 wt %, at 298 K/1.00 bar) [45] or the melamine-based microporous PAN-NH-NH_2_ (9.7 wt %) [34]. There was no saturation observed when the pressure reached to 1 bar, indicating that a higher CO_2_ capacity could be obtained by further increasing the pressure. The isosteric heat (Q_st_) of each polymer was calculated based on the adsorption data at different temperature using the Clausius–Clapeyron equation (Supporting Information File 1, Figure S7). At the zero CO_2_ gas surface coverage, the limiting enthalpies of adsorption of the three samples was similar (**P3**:30 kJ/mol, **P10**:28 kJ/mol, **P11**:29 kJ/mol) and within the scope of physical adsorption [46], which was beneficial to the materials reuse.

**Table 4:** CO_2_ adsorption of **P3**, **P10**, and **P11**.

Samples	CO_2_ uptake [cm^3^ g^−1^]		Q_st_ [kJ mol^−1^]
	273 K	298 K	

**P3**	48.5	29.7	30
**P10**	50.3	31.2	28
**P11**	52.4	32.2	29

## Conclusion

Using the Friedel–Crafts reaction, 9-PCz microporous polymers (**P1**–**P11**) were prepared by varying the molar ratio of cross-linker to monomer (**P1**–**P5**), the reaction temperature *T*_1_ (**P3**, **P6**, and **P7**), the amount of catalyst used (**P3**, **P8**, and **P9**) and the concentration of reactants (**P3**, **P10**, and **P11**). The systematic study showed that the molar ratio of cross-linker to monomer was the main way to influence the BET specific surface area. A sufficient amount of cross-linker was the premise of a superior BET specific surface area. Increasing the reaction temperature *T*_1_ or the amount of catalyst used could increase the pore volume greatly but sacrificed in part the BET specific surface area. Changing concentration of reactants could remedy this situation. When slightly varying the concentration of reactants simultaneously, a high surface area and high total pore volume could be obtained. Those provided a reference for preparing HCPs using Friedel–Crafts polymerization. The BET specific surface area of the prepared HCPs was up to 769 m^2^ g^−1^, and the CO_2_ uptake capacity was up to 10.4 wt % at 273 K/1 bar.

## Experimental

### Materials

9-Phenylcarbazole, FDA and DCE were purchased from Aladdin Chemical Reagent Corp. (Shanghai, China). FeCl_3_ were acquired from the Macklin Chemical Reagent Ltd Co. (Shanghai, China). Methanol, THF, HCl, and distilled water were obtained from TONG GUANG Fine Chemicals Company (Beijing, China). Unless stated otherwise, all solvents and chemicals were used without further purification.

#### Characterization methods

Fourier-transform infrared (FTIR) spectra of HCPs were obtained by using a Nicolet 6700 spectrometer over a wave number range of 4000–400 cm^−1^ by scanning 32 times at a resolution of 4 cm^−1^. TG analysis of the polymers were conducted with a NETZSCH TG 209F1 TG analyzer for 40–800 °C at a heating rate of 10 °C min^−1^ under a nitrogen flow of 50 mL min^−1^. The X-ray diffraction (XRD) patterns of the as prepared polymers were collected using a PANalytical X’pert Pro MPD diffractometer with Cu Kα radiation at room temperature, with step size of 0.0202°, 2θ ranging from 5.0 to 60°. Scanning electron microscope (SEM) measurements of obtained samples were carried out using a Hitachi SU1510 microscope. The nitrogen adsorption and desorption and the CO_2_ adsorption and desorption isotherms of HCPs were obtained using a GAPP V-Sorb 2800P BET surface area and pore volume analyzer. Polymers were degassed at 100 °C for over 10 h under vacuum before all gas analysis experiments.

#### Synthesis HCPs

The synthetic illustration of HCPs is depicted in Scheme 1. Using the Friedel–Crafts reaction, **P1**–**P11** have been made by changing the molar ratio of building unit to cross-linker (**P1**–**P5**), the reaction temperature *T*_1_ (**P3**, **P6**, and **P7**), the amount of catalyst (**P3**, **P8**, and **P9**) and the amount of solvent used (**P3**, **P10**, and **P11**). The synthesis of **P3** as representative procedure is given in detail: Under a nitrogen atmosphere, 9-PCz (0.67 mmol, 0.163 g), FDA (2 mmol, 0.152 g) were dispersed in DCE (18 mL) and then anhydrous FeCl_3_ (4 mmol, 0.64 g) was added to the dispersion; the mixture was allowed to react at room temperature for 5 h, then at 80 °C for 19 h with vigorous stirring. Then the mixture was cooled to room temperature and quenched by using 20 mL of CH_3_OH. Then the solid product was separated by filtration, and the solid product was washed with first methanol, followed by THF, HCl/H_2_O 2:1 (v/v) and distilled water successively, further purified by Soxhlet extraction with MeOH for 24 h and then THF for another 24 h. Finally, the product was dried in a vacuum oven at 100 °C for 24 h. The obtained polymer material was obtained as a brown solid.

The synthesis of other polymers was similar as **P3**, only the monomer amount or other experimental conditions were varied as shown in Table 1. Although washed excessively, the yield of the polymers still exceeded 100% which was due to the adsorbed catalyst or solvent in the pore structure [15]. All obtained samples were colored ranging from pale brown to dark brown.

## Supporting Information

File 1 Additional experimental results.
