# Supplementary material for: Carbazole-functionalized hyper-cross-linked polymers for CO2 uptake based on Friedel–Crafts polymerization on 9-phenylcarbazole
Source: Beilstein J Org Chem. 2019 Nov 26;15:2856–63. doi: 10.3762/bjoc.15.279 (PMC6902873; doi:10.3762/bjoc.15.279)
Supplement: File 1 — Additional experimental results. [file Beilstein_J_Org_Chem-15-2856-s001.pdf]

BEILSTEIN JOURNAL OF ORGANIC CHEMISTRY

Supporting Information

for

Carbazole-functionalized hyper-cross-linked polymers for CO_2 uptake based on Friedel–Crafts polymerization on 9-phenylcarbazole

Dandan Fang, Xiaodong Li, Meishuai Zou, Xiaoyan Guo and Aijuan Zhang

Beilstein J. Org. Chem. **2019,** *15,* 2856–2863. doi:10.3762/bjoc.15.279

Additional experimental results

License and Terms: This is a supporting information file under the terms of the Creative Commons Attribution License (http://creativecommons.org/licenses/by/4.0). Please note that the reuse, redistribution and reproduction in particular requires that the authors and source are credited.
The license is subject to the *Beilstein Journal of Organic Chemistry* terms and conditions: (https://www.beilstein-journals.org/bjoc)

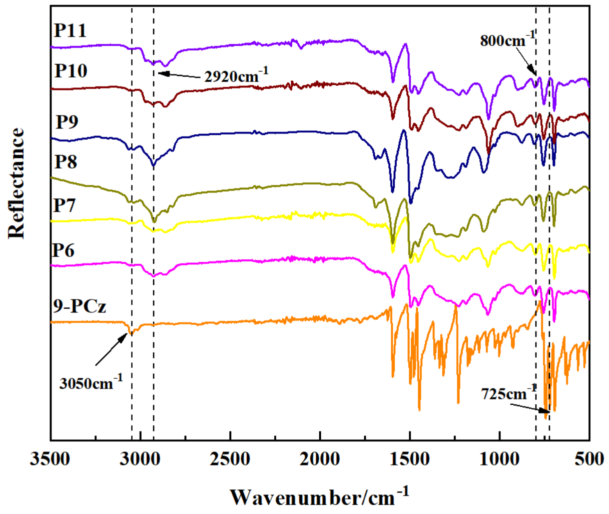

Figure S1: FTIR spectrum of HCPs **P6–P11** and 9-PCz.

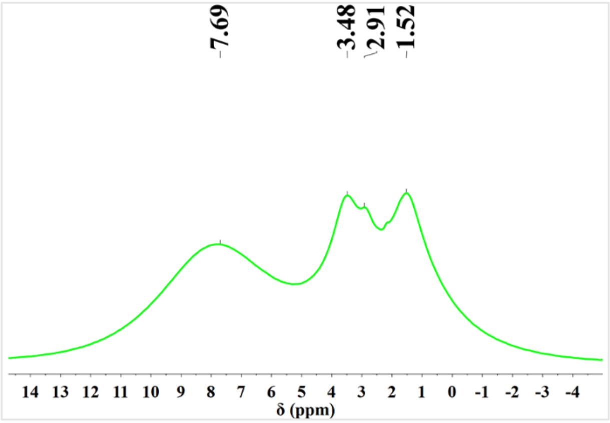

Figure S2: Solid ^{1}H NMR spectrum of **P3**.

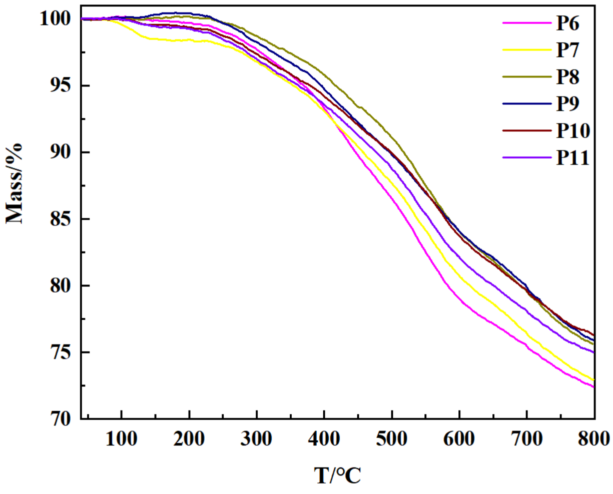

Figure S3: TGA curves of HCPs **P6–P11**.

Table S1: The thermal stability of HCPs.

HCPs	Decomposition temperature[a]/°C	Mass residues/%
**P1**	594	80
**P2**	301	74
**P3**	364	74
**P4**	274	69
**P5**	273	68
**P6**	370	72
**P7**	354	73
**P8**	417	75
**P9**	396	76
**P10**	380	76
**P11**	362	75

[a]Decomposition temperature is the 5 wt % weight loss temperature for HCPs.

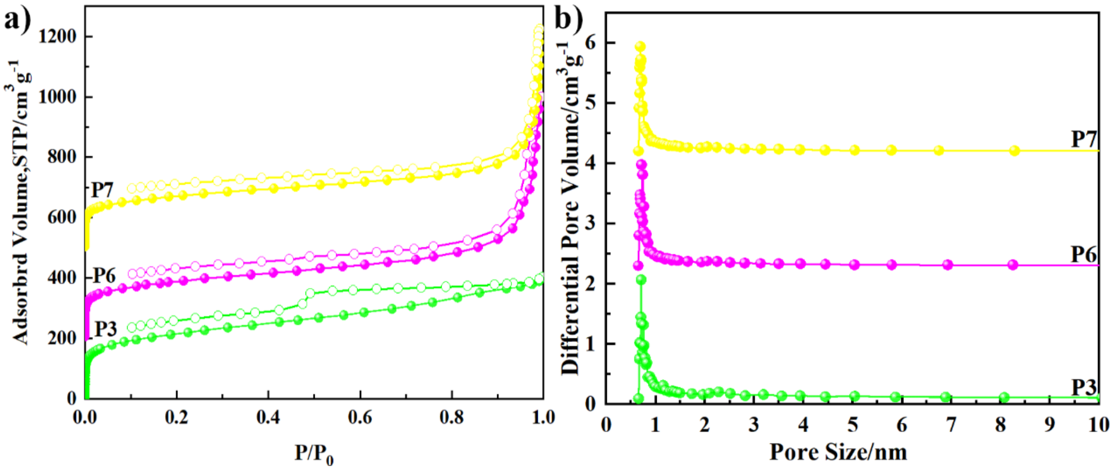

Figure S4: Nitrogen sorption isotherms (a) and pore size distribution (b) of **P3, P6, P7**.

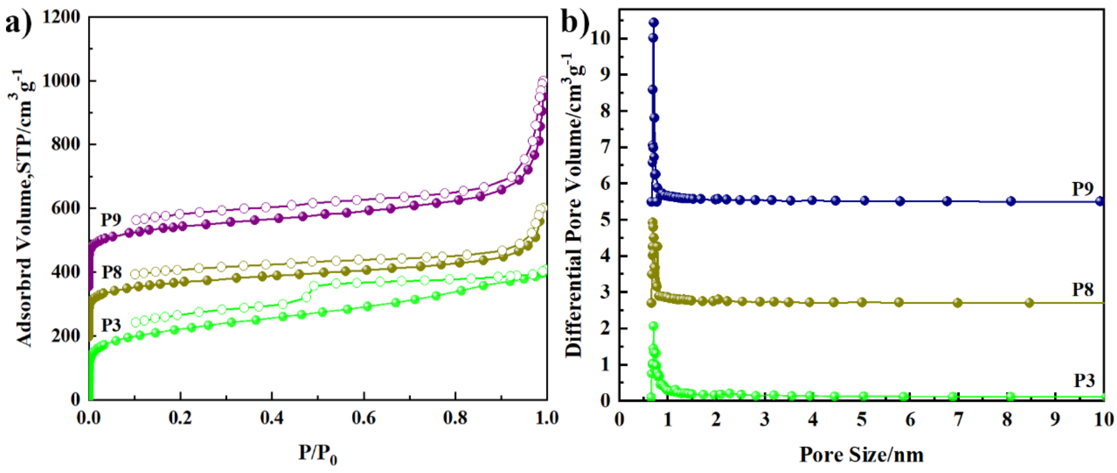

Figure S5: Nitrogen sorption isotherms (a) and pore size distribution (b) of **P3, P8, P9**.

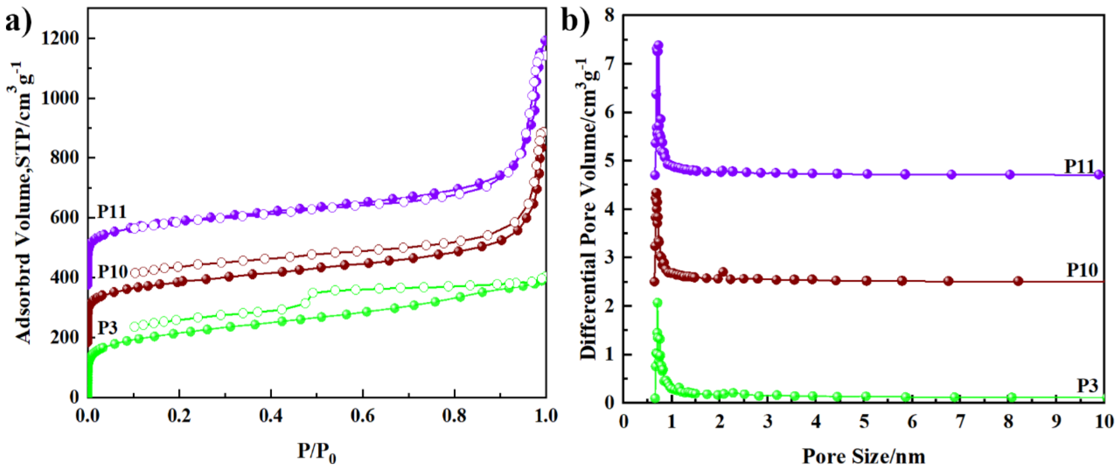

Figure S6: Nitrogen sorption isotherms (a) and pore size distribution (b) of **P3, P10, P11**.

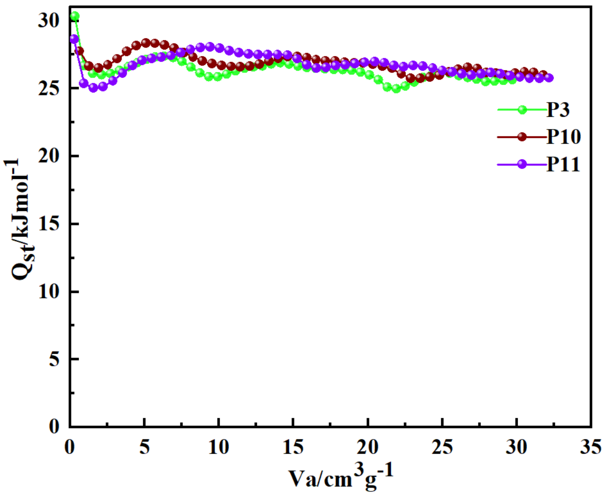

Figure S7: the isosteric heat curves of **P3**, **P10**, **P11**.
